# Digital Health in Physicians' and Pharmacists' Office: A Comparative Study of e-Prescription Systems' Architecture and Digital Security in Eight Countries

**DOI:** 10.1089/omi.2020.0085

**Published:** 2021-02-10

**Authors:** Bader Aldughayfiq, Srinivas Sampalli

**Affiliations:** Faculty of Computer Science, Dalhousie University, Halifax, Canada.

**Keywords:** e-prescription, digital security, privacy, system architecture, digital authentication, digital health, Blockchain

## Abstract

e-Prescription systems are key components and drivers of digital health. They can enhance the safety of the patients, and are gaining popularity in health care systems around the world. Yet, there is little knowledge on comparative international analysis of e-Prescription systems' architecture and digital security. We report, in this study, original findings from a comparative analysis of the e-Prescription systems in eight different countries, namely, Canada, United States, United Kingdom, Australia, Spain, Japan, Sweden, and Denmark. We surveyed the databases related to pharmacies, eHealth, e-Prescriptions, and related digital health websites for each country, and their system architectures. We also compared the digital security and privacy protocols in place within and across these digital systems. We evaluated the systems' authentication protocols used by pharmacies to verify patients' identities during the medication dispensing process. Furthermore, we examined the supporting systems/services used to manage patients' medication histories and enhance patients' medication safety. Taken together, we report, in this study, original comparative findings on the limitations and challenges of the surveyed systems as well as in adopting e-Prescription systems. While the present study was conducted before the onset of COVID-19, e-Prescription systems have become highly relevant during the current pandemic and hence, a deeper understanding of the country systems' architecture and digital security that can help design effective strategies against the pandemic. e-Prescription systems can help reduce physical contact and the risk of exposure to the virus, as well as the wait times in pharmacies, thus enhancing patient safety and improving planetary health.

## Introduction

Ensuring the safety of patients is one of the primary goals of all health care services. Most of these services rely on health information technologies related to the patient. Unfortunately, the availability of information about patients is often not adequate. Therefore, developing technologies that support medical decisions to provide quality care for patients is a necessity. Researchers proposed new approaches and technologies for managing patients' medical data and benefit from the medical history of patients to provide better medical care. The technologies were motivated by the lower efficiency of traditional methods in collecting and providing this information.

The interest of digital health and related technologies such as machine learning (ML) increased rapidly in clinical medicine as well as biomedical research and drug discovery (Koromina et al., 2019; Swan et al., 2013). Electronic Health Records (EHRs) are another and critical component of digital health, which help enhance patients' health care by transforming medicine from analog to digital age (Birkhead et al., 2015; Motulsky et al., 2015; Ploner and Prokosch, 2020; Shickel et al., 2018). Although the technology for creating patients' EHRs is advancing, records are still not available for caregivers and visiting patients from other health centers (Motulsky et al., 2015).

Medication errors can be a cause of significant concern to patient health. These errors can occur at any stage of the medication prescribing or dispensing process. They can occur when a prescription created for a medication that interacts with another medication the patient is taking or causes an allergic reaction. Moreover, errors can occur at the pharmacy due to the misinterpretation of paper prescriptions because of handwriting or missing information (Aldughayfiq and Sampalli, 2018; Nair et al., 2010; Samadbeik et al., 2017; Velo and Minuz, 2009).

Hence, e-Prescription systems can ensure patient safety while prescribing medication and are gaining popularity (Agrawal, 2009; Porteous et al., 2003). One of the benefits of e-Prescription is to improve the quality-of-care service and patient safety by reducing medication-prescribing errors (Agrawal, 2009). Moreover, a study about transferring prescriptions electronically was conducted in the United Kingdom with focus groups, and interviews with participants from all the involved parties, that is, patients, general practitioners, and pharmacies, after UK's National Health Service (NHS) revealed their intention to use the e-Prescription system. The study found that using e-Prescription will enhance patients' convenience, especially for patients who have repeated prescriptions (Agrawal, 2009; Deetjen, 2016; Porteous et al., 2003).

E-Prescription is defined as using an electronic device to submit and exchange the prescription information among the involved parties, namely, the patient, prescriber, pharmacy, and health insurance company. It is worth mentioning that the patient involvement in the majority of the e-Prescription systems we reviewed is only to consent to use an e-Prescription by the prescriber and the pharmacy. The use of e-Prescription will allow the involved parties to provide a safe, quality, and efficient care service. Moreover, e-Prescription systems will provide the communication medium between a prescriber and a pharmacist upon reviewing a prescription before dispensing (AMA et al., 2011; Bell et al., 2004; Mon, 2009; Odukoya and Chui, 2013; Samadbeik et al., 2017; Van Dijk et al., 2011).

E-Prescription will likely reduce medication errors caused by paper prescriptions. In addition, e-Prescription will improve the low service quality associated with paper prescriptions by decreasing the amount of work needed to sort the related paperwork. More importantly, providing a medication history for each patient will enhance the patient safety while prescribing medication (Aldughayfiq and Sampalli, 2018; Byrne et al., 2010; Devine et al., 2010; Kohn, 2011; Odukoya and Chui, 2013; Samadbeik et al., 2013; Taylor et al., 2008; Timonen et al., 2018; Van Dijk et al., 2011; Wang et al., 2009). However, not all medication errors are entirely preventable by e-Prescriptions. Moreover, there are risks related to the prescriber's adaptation of the e-Prescription system, since they need to familiarize themselves with the e-Prescription software (Odukoya and Chui, 2013; Timonen et al., 2018).

In addition, according to one study, nearly 5% of e-Prescriptions introduced errors related to the prescriber's information entry or due to a lack of information about the appropriate treatment procedure (Odukoya et al., 2014). Discovering these risks is more likely to eliminate them if found by the pharmacist or by including more features in the system. These features will support the prescriber's decision to the benefit of patients' safety (Odukoya et al., 2014; Reed-Kane et al., 2014; Salmon and Jiang, 2012; Yang et al., 2018).

The COVID-19 pandemic has made e-Prescription systems especially relevant. Physical distancing, limiting unnecessary trips out of home, and minimizing social contacts have become necessary worldwide (WHO, 2020). E-Prescription systems is likely to help in reducing visits to the clinics for picking up prescriptions and reduce the wait times in pharmacies when prescriptions are sent electronically in advance for medications to be prepared. Moreover, implementing ePrescription will minimize the risk of getting exposed to the virus due to handling paper prescriptions.

We report here original findings from a comparative analysis of the e-Prescription systems in eight different countries, namely, Canada, United States, United Kingdom, Australia, Spain, Japan, Sweden, and Denmark.

We explore recent studies conducted in the domain of digital health and e-Prescription systems. Wherever available, an overview of the digital security and privacy protocols in place for each e-Prescription system is highlighted. Furthermore, we discuss the protocols and policies for verifying patient identity. We identify the challenges in the current systems drawing from the comparative analysis and solutions are suggested as well.

This study critically compares the currently implemented e-Prescription systems in the selected countries and evaluates the security and privacy protocols of those systems and the capability of those systems to integrate new technologies such as Artificial intelligence (AI) and Blockchain.

## Materials and Methods

We have reviewed and explored e-Prescription systems using a jurisdiction comparison method. Countries with e-Prescription system were selected from each content.

The selection process was as follows:

(1) We chose the leading countries that have deployed e-Prescription systems from each continent. In Europe, many countries have adopted digital health initiatives in the past decade. However, we considered a few leading countries that have fully implemented e-Prescription systems. This approach is part of the national electronic-health strategy in the European Union (EU) countries (AEPI eHealth Initiative, 2004; Johnston et al., 2003).
(2) In the second stage, we considered the availability of the e-Prescription systems in community pharmacies and whether the system is nationwide or state/province-wide in the selection process. We excluded e-Prescription implemented only within hospitals or health centers.
(3) A key factor in our selection process is the security and privacy protocols, which we used to compare and assess the e-Prescription systems from a technical and security aspect.

Finally, the countries resulted from the selection process were four EU countries (United Kingdom, Spain, Sweden, and Denmark), two North American countries (United States and Canada), Australia, and Japan.

The data collection process was based on the main components of the e-Prescription system model (eHealth Initiative and Center for Improving Medication Management, 2008; eHealth Observatory, 2011; Samadbeik et al., 2017; The Center for Improving Medication Management, 2011). The publicly available data collected from the countries included the following:

- The e-Prescription system architecture components: Such components are the architecture type (i.e., centralized or decentralized system), prescription database, medication database, medication history database, clinical decision support (CDS) features, issuing a paper prescription, electronic prescribing types, medical records, and e-Prescription for controlled medicine.
- The system security and privacy protocols (use of Health Level Seven International [HL7] protocol, patient consent, and patient's identity verification) and the system components identifiers (Pharmacy ID, Prescriber ID, Medication ID, Prescription ID, and Patient ID).
- The e-Prescription system process (the e-Prescription information availability to the involved parties, the availability of Drug-Drug Interactions [DDI] information based on the patient health record, storing the e-Prescription information for future uses, and the electronic transfer of the prescription to a pharmacy).

Data for this survey were retrieved by searching for keywords and/or a combination of keywords from the search engines Google, Google Scholar, PubMed, IEEE, ACM, Dalhousie University Libraries, and the official digital health websites of the selected countries.

The keywords used for the search were “Eprescription,” “e-prescription,” “electronic prescription,” “e-Rx,” “eDispensing,” or “electronic dispensing” with the name of each of the selected countries. Then, all the retrieved papers and related documents were examined. In addition, we compared all the retrieved data with the official website of the systems used in this survey to remove any outdated or false information. Finally, we compared the systems' countries, and the data are shown in comparative tables.

## Results

### e-Prescription systems

#### PrescribeIT: Canada's e-prescription system

PrescribeIT is a government-founded system for e-Prescriptions. The system has been partially implemented in some of the provinces and entirely in others. The system's aim is to be used across the nation in all the provinces in the near future. Infoway conducted a workshop in 2016 with a number of prescribers and pharmacists to explore issues in the paper prescription system (Canada Health Infoway, 2018). Therefore, the system's main purpose is to act as a medium to transfer and exchange prescription information between a prescriber and a pharmacist. The following are the main requirements that resulted from the study for PrescribeIT (Nayani, 2017):

- Secure communication between the pharmacy and the prescriber.
- Effective Drug Information System (DIS) to detect drug interactions for both the pharmacy and prescriber.
- Integration with an Electronic Medical Record (EMR) management system.
- e-Prescription status and alert to the prescriber.
- Security and privacy in accessing patient information.

PrescribeIT defines e-Prescription as the process of transmitting a prescription between a prescriber and a pharmacy with the condition of not affecting the clinical workflow (Canada Health Infoway, 2018). Therefore, PrescribeIT's primary focus is to enable transmitting an e-Prescription securely between the involved parties. In addition, PrecribeIT met the requirements by integrating the system with existing health care systems (e.g., DISs, and EMR) available in care provider software (Green and Reinholdt, 2017).

**FIG. 1.**
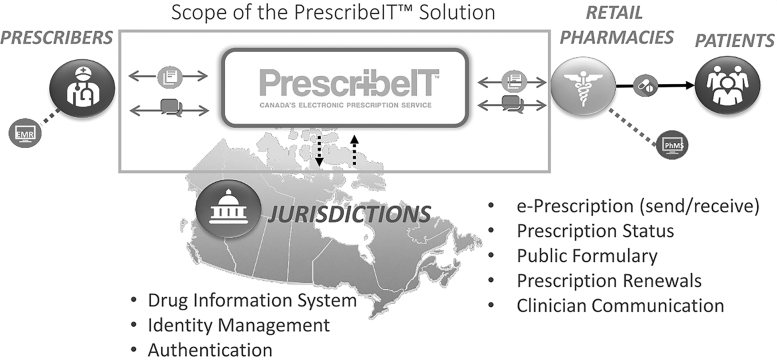
PrescritbeIT overall structure (used with the permission of Canada Health Infoway, 2018).

**FIG. 2.**
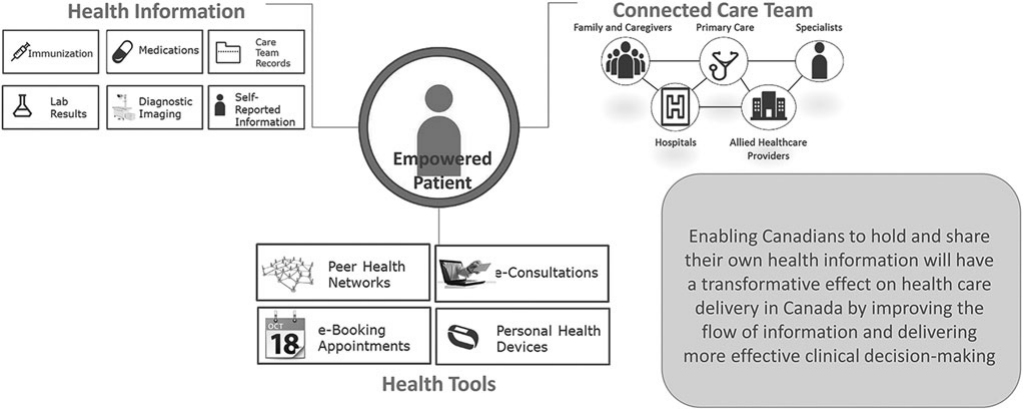
PrescribeIT future features (used with the permission of Canada Health Infoway, 2018).

The prescription information is sent encrypted from a prescriber to a patient's pharmacy of choice. Moreover, in terms of security, the system provides access control. Figure 1 illustrates the architecture of the system. PrescribeIT aims to connect the involved parties by enabling them to exchange prescription information. The system intends not to replace the current management system in the pharmacies or the prescriber's office. Instead, the system helps monitor the prescription by storing the prescription information of a patient in the system. Figure 2 shows the complete architecture and features that will be deployed in the future.

#### Patients' data security and privacy

The prescription information is sent encrypted from a prescriber to a patient's pharmacy of choice. Moreover, the user of PrescribeIT (i.e., a prescriber or a pharmacist) must use multifactor authentication to access the patient's prescription information. An access control process is used to grant and revoke accounts on the system. The user is required to use password authentication to access the assigned levels in the system. Moreover, for security, all transactions in the system are logged and audited (Canada Health Infoway, 2019; PrescribeIT, 2018).

#### Surescripts: United States e-prescription system

**FIG. 3.**
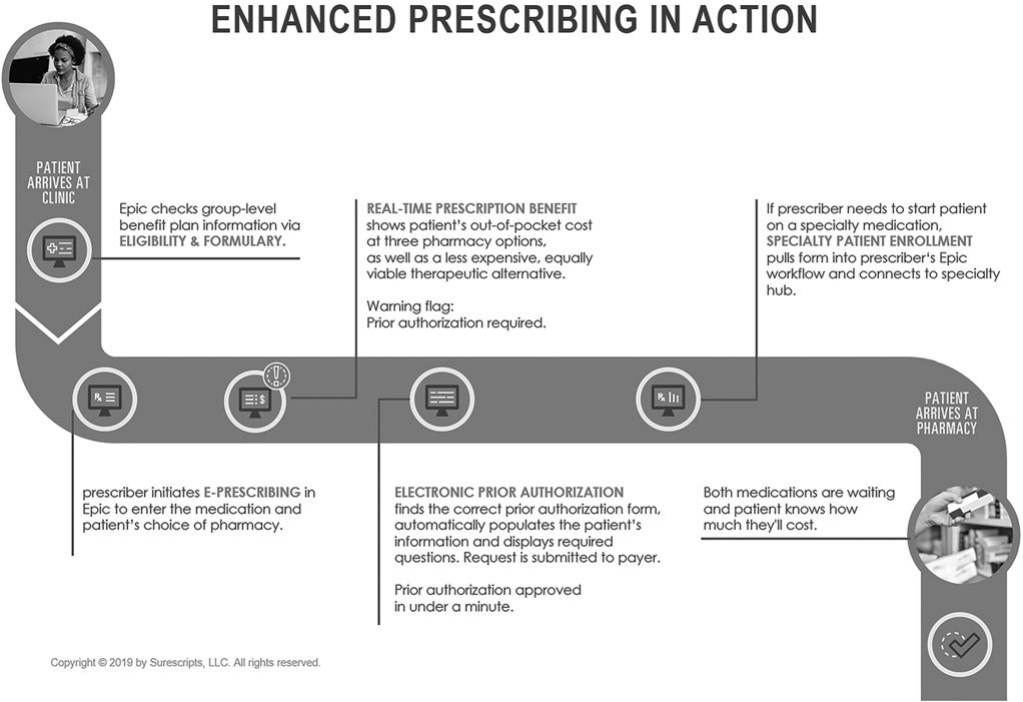
Key features of the Surescripts system (used with the permission of Surescripts, 2019).

Surescripts is an e-Prescription network where the stakeholders in the system can communicate and exchange data. Surescripts is a decentralized e-Prescription network. The parties in the network can communicate with each other using peer-to-peer communication (Surescripts, 2018a). Surescripts provides the prescriber with the patient's medication history and formulary and benefits information from participating insurers and pharmacy benefit managers (PBMs) (Castro, 2009; Joy et al., 2011; King et al., 2007). Figure 3 illustrates the key features of the Surescripts system.

#### Patients' data security and privacy

Surescripts manages the security and privacy of the patient data based on the provided service. Benefit optimization is one of the services that Surescripts provides to caregivers. This service ensures that the patient's drug information is updated and accessible in real-time during patient visits. Surescripts works with the PBMs and the health care payers to acquire this information. Another service Surescripts provides is the medication history.

This service provides the caregivers with medication-related information about the patient from the participating patient's community pharmacies and health insurance companies. This service requires the patient's consent to give the caregivers access to the patient's medication history information. Clinical history is another service provided by Surescripts. In this service, the caregivers will request the previous care location the patient has attended. The service will cover the location of the past health record and the past prescribed and dispensed prescriptions. Surescripts handles the caregiver request for the medical record from the discovered location about the patient. Most importantly, the e-Prescription service allows the exchange of the prescription electronically. The network allows the prescriber and the pharmacy to exchange prescription information (Surescripts, 2019).

#### Electronic prior authorization

A prescriber asks for prior authorization (PA) from a patient's health insurance before prescribing any medication. This requirement is the health insurance technique used for minimizing the cost of covered medications. In addition, the insurance will not pay any benefits for any medical care without preapproval. However, this is mostly the case for more expensive medication. Several drugs are subject to PA. The following is a list of the most frequent reasons why PA is required (Gasbarro, 2015):

- Brand medications that are available in a generic form
- Expensive medications
- Cosmetic medications
- Medications not usually covered by insurance companies.

Obtaining PA used to be a challenging process. In the past, prescribers needed to send the prescription to the pharmacy choice of the patient. Then, the pharmacist would start to process the prescription and find out if the prescription needed a PA, usually through a phone call or by faxing a form. The patient would then be informed using the available channels, usually by phone. Following that, the pharmacist would start the PA approval process using phone calls or fax. This process would take days or weeks to finish.

**FIG. 4.**
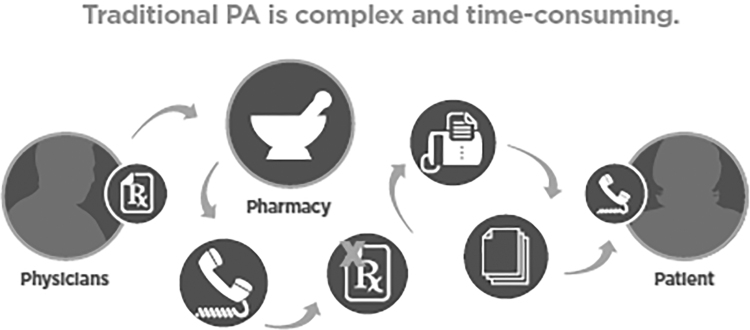
Traditional PA (used with the permission of Surescripts, 2019). PA, prior authorization.

Finally, after getting approval, the patient would be notified through a phone call that the prescription is ready to be picked up. In addition, the increased use of expensive drugs that require PA approval made the process more complicated and time consuming. The process of obtaining PA eventually affected the quality of service at the prescriber's office. Finally, the prescriber's office had to meet all the different requirements from the insurance, based on the plan and the patient (Surescripts, 2015). The PA approval process sometimes would take several days. According to Surescripts, 69% of the patients had to wait several days to get their medications approved by the insurance company (Surescripts, 2015). Figure 4 illustrates the traditional process of PA.

**FIG. 5.**
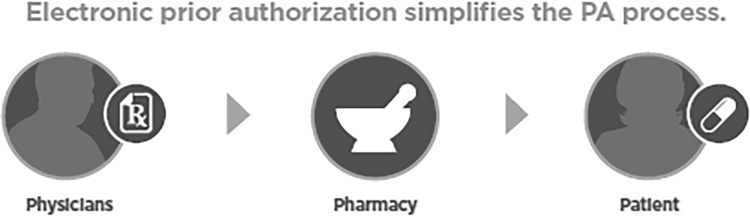
Electronic PA in Surescripts (used with the permission of Surescripts, 2019).

Surescripts provides an ePA process. This process simplifies the process and increases the efficiency of getting the prescription from the pharmacy without any delay. The prescriber will request the PA approval during the e-Prescribing process. The system will notify the prescriber if there is a PA requirement or not. Then, the prescriber has the option of selecting another medication option or sending PA electronically using the EHR system. Following this, the prescription will be sent to the pharmacy, where it will be ready to be picked up (Surescripts, 2015). Figure 5 illustrates the electronic PA process.

#### Australia's e-prescription system

The Australian Digital Health Agency defines electronic prescription as an Electronic Transfer Prescription (ETP) service. ETP is defined as transferring a prescription securely between a prescriber and pharmacy. The pharmacies and prescribers must use a Prescription Exchange Service (PES) system to communicate and exchange the prescription information securely. The PES system must be approved by the Commonwealth and meet specified security and privacy standards. In Australia, there are currently two PES systems: electronic medical prescription (eRx) Script Exchange and MediSecure. The involved parties (i.e., the pharmacy or the prescriber) may be connected to one or more PES systems.

According to the Australian Digital Health Agency, the prescriber is responsible for registering their clinical practice with a PES. Also, the prescriber must have software with the ability to send e-Prescriptions. Moreover, the prescriber is responsible for encryption key management. The e-Prescription must be encrypted when transferred to the pharmacy's PES. Moreover, both ETP and PES services are essential components for keeping records of the prescriptions and dispensing history.

**FIG. 6.**
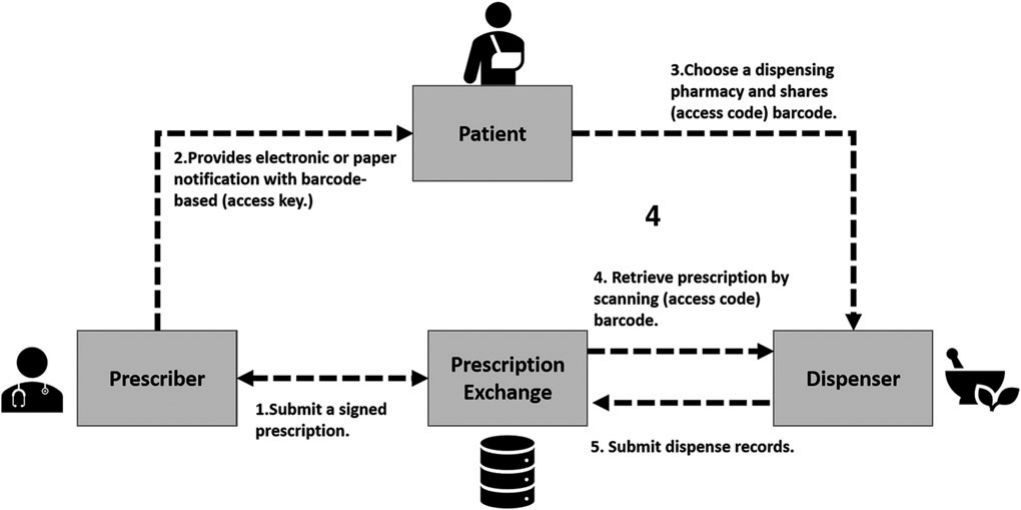
Australia eRx Architecture (adapted from eRx, 2018a). eRx, electronic medical prescription.

The records are stored in the patient's health record in the My Health Record system. Then the prescription and dispensing information can be viewed through the system. For that, the provider and the pharmacy must have the patient's consent to upload the information to the My Health Record system, and the patient must have an active My Health Record account. The authorized health care providers can view prescription and dispensing history through My Health Record system (The Australian Digital Health Agency, 2019a, 2019b). Figure 6 illustrates the Australian eRx architecture.

eRx meets all the legal privacy requirements described in the Privacy Act 1988 in Australia and the eAuthentication framework of the Australian Government (2009; eRx, 2018a). According to eRx, all the prescription information is encrypted when transferred through the system. eRx acts as an electronic mail carrier, and only the prescriber and the pharmacist can access the prescription information (eRx, 2018a). eRx can only unlock the first layer of the three-layer encryption. The first layer has just the header information of the data package. This information is needed to send the right prescription corresponding with a scanned barcode in the paper prescription. The header information does not include any personal or medical information about the patient (eRx, 2018b).

MediSecure offers the same service as eRx in terms of being an electronic medium used to transfer prescription information between the involved parties. In addition, MediSecure offers the DrShop service, which is a real-time prescription monitoring service. This service will provide the prescriber alerts, if the prescribed medication could lead to addiction (MediSecure, 2019a). In terms of privacy, MediSecure (2019c) follows the same privacy methods as eRx. However, MediSecure (2019c) has a secure Script Vault, where they will keep the encrypted prescription until it is retrieved by the pharmacy. Moreover, patient consent is required to send the prescription electronically through MediSecure (2019b).

#### United Kingdom's e-prescription system

According to the United Kingdom NHS, almost 1.5 million prescriptions are processed every day, and this rate is expected to increase by 5% every year. Seventy percent of those prescriptions are repeat prescriptions. Therefore, to provide more efficient and accurate service, electronic prescriptions are necessary (NHS BSA, 2018). The NHS identifies that the most common users of the Electronic Prescription Service (EPS) are patients who get repeat prescriptions and patients who use one pharmacy to dispense all their prescriptions (NHS, 2019).

**FIG. 7.**
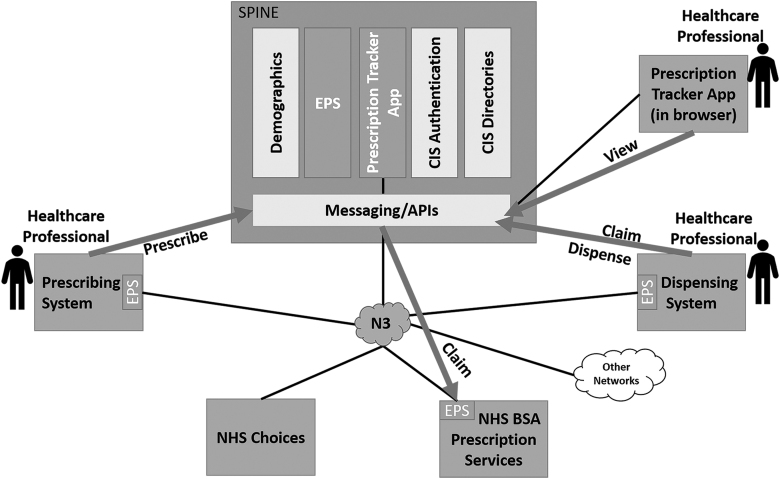
UK e-Prescription service architecture (adapted from NHS, 2019).

Furthermore, EPS is a more efficient method to send prescriptions securely to pharmacies. The EPS is sent through the NHS Spine system. Spine is a central system that allows the secure exchange of patients' health and care information between care provider organizations when needed (National Health Service Digital, 2019e; PSNC, 2019). Patient consent is needed for participation in the EPS. Figure 7 shows the EPS overview system (National Health Service Digital, 2019c).

The system uses smartcard authentication for the health care provider to access NHS Spine services, such as EPS and the patient's Summary Care Record (National Health Service Digital, 2019d, 2019e; PSNC, 2019). Spine has more than 800,000 Smartcard users. The service is used to identify the health care provider and their access levels for patient information (National Health Service Digital, 2019d; PSNC, 2018). The system also provides the ability to choose the preferred pharmacy for the patient through the prescriber. This step is called nomination, and a patient's consent is required to participate in the EPS service. Moreover, the patient has the right to request a paper prescription at any time from the prescriber (Hibberd et al., 2017; NHS, 2019; PSNC, 2016).

Moreover, The system uses unique identifiers for the prescription form, and when the prescriber issues a prescription, the system creates three identifiers: (1) the prescription form, (2) the short prescription form ID, and (3) the prescription line item Unique User Identifier (UUID). Identifiers 1 and 3 will not be visible for the end users and only used by the messaging protocol HL7 (HL7, 2019; HL7UK, 2019). Identifier number 3 will be visible to the end users and printed and barcoded in the paper prescription (Hibberd et al., 2017; National Health Service Digital, 2019b). NHS has allowed the use of EPS to prescribe a selected list of controlled drugs as of March 25, 2019. For the controlled drugs not on the selected list, the prescriber will need to use paper prescriptions (National Health Service Digital, 2019a).

#### Spain's e-prescription system

In Spain, the e-Prescription system's primary goal is to ensure the patient's safety and improve the patient's treatment care. According to the health authorities in Spain, the system must include a list of possible medications that allowed to be prescribed. The medication list has a coding system for all the information about every medication approved on the list. The list is likely to help detect drug interactions.

**FIG. 8.**
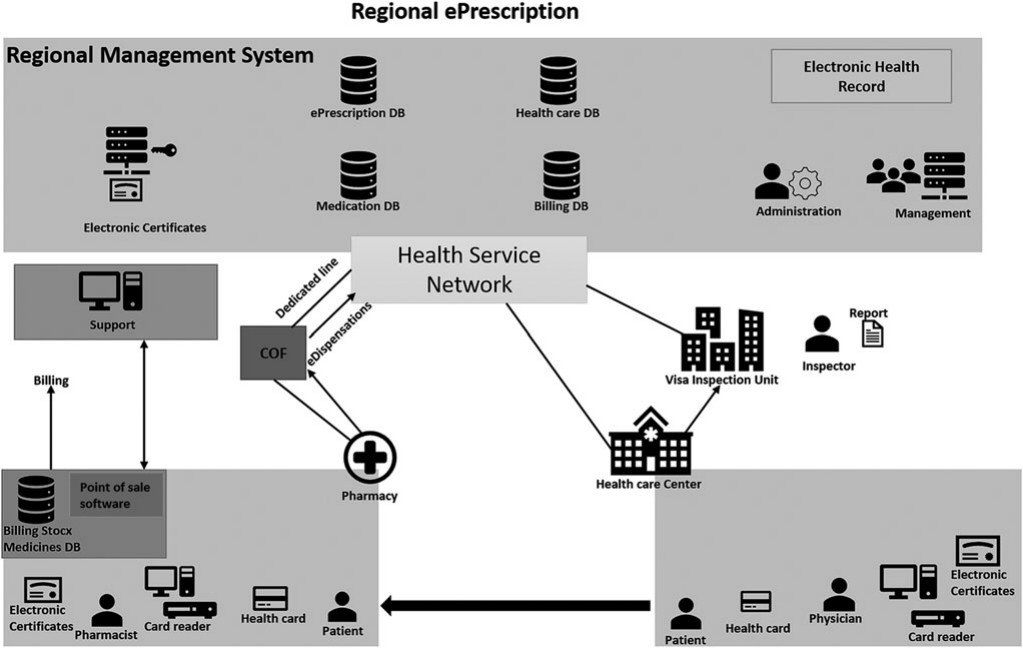
Spain e-Prescription system architecture (adapted from Ministry of Health, Social Services, and Equality, 2014).

Moreover, the system is connected to the patient's EHR to help identify any additional other interactions or allergies to the prescribed medicine. In addition, the prescription will be shared with any other prescriber treating the patient. Furthermore, the active prescription will be accessible by all pharmacies in the country. The patients will be able to pick up their medications at any pharmacy in the country or in the surrounding countries using the eDispensation service, which is part of the e-Prescription system. Finally, the system uses Systematized Nomenclature of Medicine-Clinical Terms (SNOMED-CT) to code all the information in the system (Kierkegaard, 2013; Ministry of Health, Social Services and Equality, 2014). Figure 8 illustrates the Spanish ePrescribing system architecture.

#### Japan's e-prescription system

**FIG. 9.**
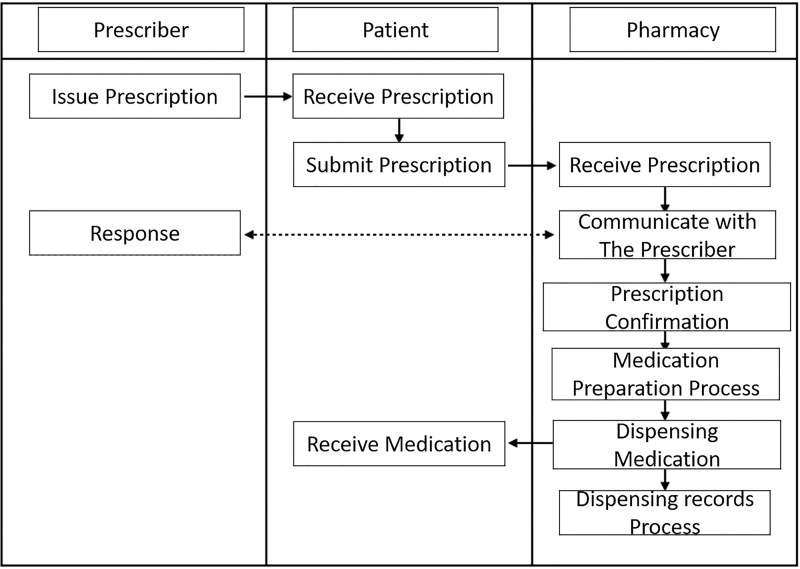
Japan current prescription process steps translated from Ministry of Health, Labor, and Welfare (2019).

The current prescription dispensing process in Japan is still in paper form. Figure 9 shows the flow of the dispensing process. The prescriber prints the paper prescription and delivers it to the patient. Then, the patient submits the prescription to the pharmacy of their choice. Next, the pharmacy starts the process of dispensing the medication and dispenses it to the patient. Finally, the pharmacy prepares the medication-dispensing records.

In addition, patients in Japan have a notebook where they keep a sticker for each dispensed medication. The pharmacy provides the stickers after dispensing. Some of the pharmacies provide an app that acts as the medication history notebook. This notebook acts as a medication database for each patient (Japan Government, 2019; Ministry of Health, Labor and Welfare, 2019; Nakagawa and Kume, 2017). Even though Japan is using a paper prescription format, the government has proposed electronic prescription system guidelines in 2016 (Akiyama and Nagai, 2012; Masuda, 2016; Ministry of Health, Labor and Welfare, 2016).

**FIG. 10.**
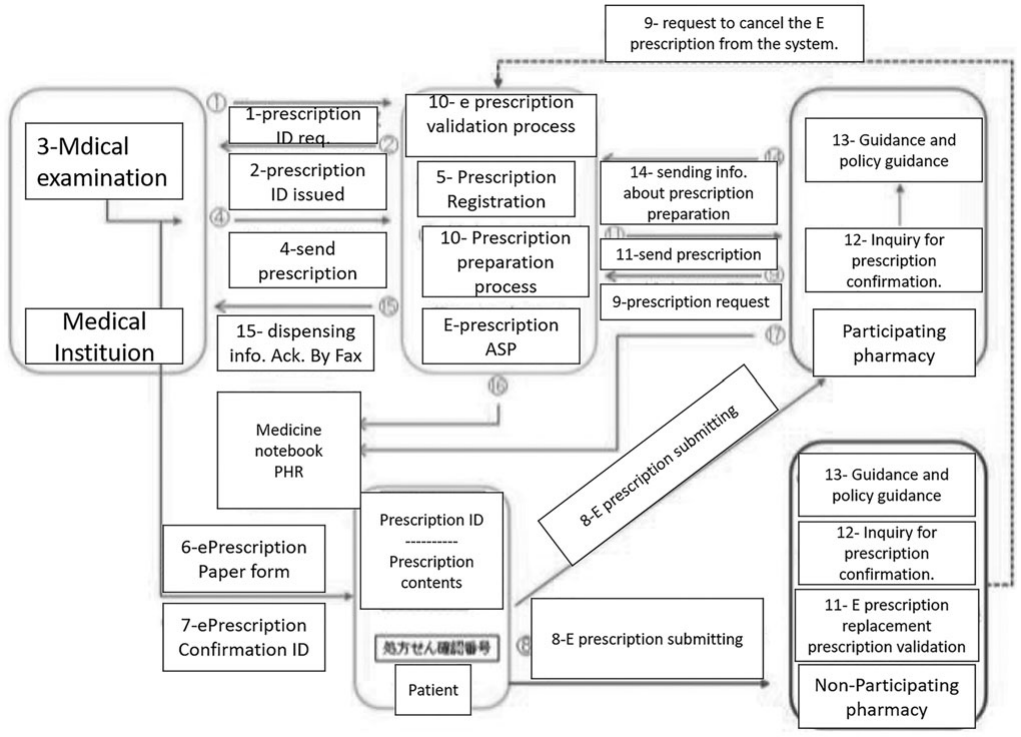
Japan e-Prescription system in the 2016 guidelines (translated) (Ministry of Health, Labor, and Welfare, 2016).

Figure 10 shows the flow as described in the guidelines published in 2016. The system proposes the use of a copy of the electronic prescription in a paper form. The electronic prescription paper contains the prescription ID with the prescription contents. This version of the electronic prescription is carried by the patient and submitted by hand to the pharmacy. There are two types of participating pharmacies in this system. A pharmacy equipped with a management system that can handle electronic prescriptions. The second type is pharmacies, where only the paper version of the prescriptions is acceptable (Japan Government, 2019; Ministry of Health, Labor and Welfare, 2016).

The Health Ministry in Japan later conducted interviews with the involved parties, namely, prescribers and pharmacies. The result of the interviews is that the proposed system is more complex and requires the added cost of hiring more staff to manage different system components. Therefore, as a result, they proposed more simplified system guidelines, which were supposed to be ready for use in late 2019 or early 2020 (Ministry of Health, Labor and Welfare, 2019).

**FIG. 11.**
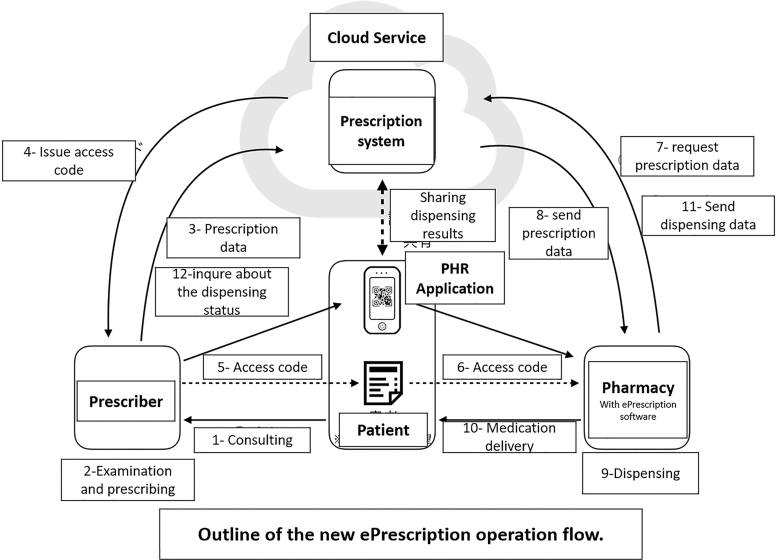
Japan new e-Prescription system expected in 2020 (translated) (Ministry of Health, Labor, and Welfare, 2019).

Figure 11 illustrates the newly proposed system where the patient gets an access code from the prescriber. The prescription system issues this access code after the prescriber submits prescription data. The patient can choose to get the access code in a paper form or an electronic form sent to their Personal Health Record (PHR) application. The system generates the access code using QR code technology. After the patient goes to the pharmacy to pick up the medication, the pharmacy scans the QR code to get the prescription information from the prescription system in the cloud. The pharmacy then starts the dispensing process. Finally, the pharmacy updates the prescription system with the prescription dispensing data. Furthermore, the patient's PHR application will be updated with the dispensing information to keep it in the electronic medication notebook (Japan Government, 2019; Ministry of Health, Labor and Welfare, 2019).

According to the Distribution news (a Japanese news website), the Ministry of Health in Japan published an official statement about its final report on the e-Prescription system design study results in March 2019 (Japan Government, 2019). The system will connect the EMR system with the pharmacies' databases using the HL7 standard Fast Healthcare Interoperability Resources (FHIR) (HL7, 2019; HL7-FHIR-Release-4, 2019; Japan Government, 2019; Ministry of Health, Labor and Welfare, 2019).

#### e-Prescription overview in Sweden

The computerization of Sweden's health care started in the 1970s when the National Corporation of Swedish Pharmacies was the only pharmacy retailer in Sweden. They distributed minicomputers to all the offices in Sweden with built-in software from the Swedish branch of Data General. These minicomputers printed medication labels to simplify safety checks in the pharmacies and at the patient's home. In addition, the minicomputers played an essential role in developing the national prescription database in the early years of e-health compared with other countries.

In the 1980s, patient smart cards were introduced to replace paper prescriptions. The patient's smart cards contain information about recently prescribed medications. After the prescriber writes the information on the card, the patient takes it to a pharmacy. Then, the pharmacist can access the information in the card with the help of the supporting system. Furthermore, the patient can take the card, which holds their recent medication history, to any other prescriber. In the prescription writing process, the prescriber uses the support system to access all the information about medication from a national database generated from three sources:

- The product database created and updated by the pharmacies.
- The medication information about each medication, the recommended dose, and the side effects.
- The drug book is containing information about diseases and which medications are used to treat certain diseases.

For access control, the smart card developers made the patient's information only accessible by using the keys stored in the authorized caregiver card keys. In the late 1990s, the use of EHR systems in outpatient clinics increased by 90%. Therefore, interest in the electronic transfer of prescriptions has greatly increased in recent decades. Sweden and Denmark were the world leaders in the adoption of electronically transferring prescriptions using the Electronic Data Interchange For Administration Commerce and Transport (EDIFACT) message format. In 2001, the message format was replaced by the XML message format based on the European pre-standard ENV 13607.

**FIG. 12.**
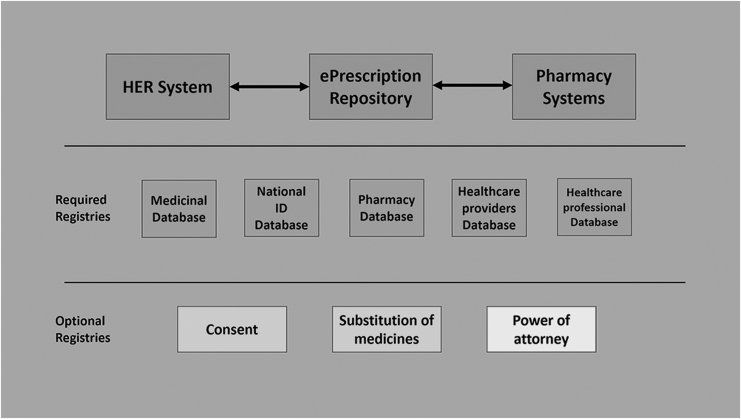
Sweden e-Prescription system components (adapted from Hassel (2019).

In 2000, the National Corporation of Swedish Pharmacies replaced the process of transferring prescriptions between the prescriber and pharmacies. They requested the prescribers to electronically transfer prescriptions to an e-Prescription repository instead of using the patient's smart card. This was feasible because the National Corporation of Swedish Pharmacies was the only pharmaceutical company in Sweden. In 2019, the Swedish eHealth Agency changed the system framework by managing the e-Prescription repository. This was due to the increased number of pharmacy chains, which has led to an increased number of different systems at pharmacies (Grepstad and Kanavos, 2015; Hammar et al., 2011; Hassel, 2019; Klein, 2010; Öhlund et al., 2012). Figure 12 illustrates Sweden's e-Prescription system components.

#### Denmark's e-prescription system

Similar to Sweden, Denmark is one of the world leaders in the deployment of eHealth for the better care of patients (Hammar et al., 2011; Kierkegaard, 2013; Samadbeik et al., 2017). In the 2000s, Denmark used an ongoing EHR system accessible by all caregivers in public hospitals. Moreover, nearly 85% of Denmark's population had health records in the EHR system by the year 2011 (Krag et al., 2012). The centralized EHR system provided a robust infrastructure for establishing an e-Prescription system.

Therefore, in 2002, Denmark introduced its e-Prescription system nationwide. The Danish Medicines Agency manages the system, and the system is responsible for managing and storing the electronic prescriptions issued by a prescriber. The e-Prescriptions can then be accessed by the patient as well as by prescribers and pharmacies. The e-Prescription records, when accessed by any of the above parties, will provide an overview of all the prescribed medications (Kierkegaard, 2013; Krag et al., 2012; Samadbeik et al., 2017).

### Overall system architecture

As we can see from Table 1, the systems are divided into two types, namely, centralized and distributed. First, in centralized systems, all the medical records are stored in centralized servers that are controlled by a federal regulatory body. The centralized systems help make all the medical records for a patient in all health care centers available for the caregiver at any of the health centers. Moreover, centralized systems offer better services for future research and studies. However, many researchers and medical institutions will argue that there is a loss of patient privacy and security when using centralized systems (Zaghloul et al., 2019). Many studies showed that centralized systems are vulnerable to Distributed Denial of Service (DDoS) cyber-attacks (Zaghloul et al., 2019; Lau et al., 2000) and social engineering attacks (Zaghloul et al., 2019; Anderson, 2008; Patil and Seshadri, 2014).

**Table 1.** Comparison of the Systems' Overall Architecture

System	Surescripts	Prescritbe IT	United Kingdom	Sweden	Denmark	Spain	Australia	Japan
Benefit Optimization	✓	×	—	—	—	✓	×	×
Electronic Prescribing	✓	✓	—	—	—	✓	✓	✓
Prior Authorization	✓	×	—	—	—	—	—	×
Clinical History	✓	×	✓	—	—	✓	—	×
DDI Alerts^a^	✓	×	—	—	—	✓	—	×
Centralized System	×	✓	✓	✓	✓	✓	×	×
Prescription Database	×	—	✓	✓	✓	✓	✓	✓
Medication History	✓	✓	—	—	—	✓	Consent required	✓
Medication Database	×	—	✓	✓	✓	✓	✓	✓
Issuing Prescription	×	✓	✓	✓	×	—	✓	✓
e-Prescription for controlled Medicine	✓	—	×	×	×	—	—	×

^a^ DDI alerts incorporated as part of the system.

DDI, Drug–Drug Interaction.

Moreover, the centralized systems will limit the data privacy of the patient, as health records can be shared anywhere across the system (Zaghloul et al., 2019; Ponemon, 2018). In the US, Surescripts (Surescripts, 2015) is an e-Prescription network that helps transfer e-prescriptions between a prescriber and a pharmacist. This means the e-Prescription system is not centralized, and each part of the patient information is stored in their local system. A recent update to Surescripts provides the ability to request any health information through the network; however, both parties who want to exchange it need to subscribe to Surescripts. This means each healthcare center stores its EMR in their systems, and it is not accessible from other healthcare centers unless requested.

The decentralized systems offer more information privacy and more protection. However, the centralized approach improves the quality of the offered service and helps minimize the errors in that service. In terms of e-Prescription, one of the benefits of a centralized system is the availability of the patient's medication history to all parties. This helps minimize medication interaction errors and Adverse Drug Reactions (ADR). As shown in the US case study, decentralized systems are also able to share medication history with other parties.

However, this process is subject to in-place conditions such as a health center agreeing to share information with other parties or subscribing to the same e-Prescribing service. Other approaches, such as the Japanese, they propose that the medication history should be controlled by the patient and sent to the requesting parties (Japan Government, 2019). Moreover, other approaches provide access to the patient using web portals to display relevant information about an e-Prescription to request medication delivery to the home (Jensen and Thorseng, 2017; Kruus, 2013; Patrao et al., 2013; Sellberg and Eltes, 2017). Other researcher proposes an e-Prescription system in which the patient has the central role. This approach aims to give the patients priority in making decisions regarding their health (Pereira et al., 2018).

The decentralized systems offer more information privacy and more protection. However, the centralized approach improves the quality of the provided service and helps minimize the errors in that service. In terms of e-Prescription, one of the benefits of a centralized system is the availability of the patient's medication history to all parties. This helps minimize medication interaction errors and ADR. As shown in the US case study, decentralized systems can share medication history with other parties. However, this process is subject to in-place conditions such as a health center agreeing to share information with other parties or subscribing to the same e-Prescribing service. Different approaches, such as the Japanese, proposed that the medication history be controlled by the patient and sent to the requesting parties (Japan Government, 2019).

Moreover, other approaches suggested providing access to the patient using web portals to display relevant information about an e-Prescription to request medication delivery to the home (Jensen and Thorseng, 2017; Kruus, 2013; Patrao et al., 2013; Sellberg and Eltes, 2017). Other researchers propose an e-Prescription system in which the patient has the central role. This approach aims to give the patients priority in making decisions regarding their health (Pereira et al., 2018).

One central aspect of the e-Prescribing systems is that they support prescriber decisions regarding prescribing medications to patients. These systems aim to help prescribers safely prescribe medications to patients. Such features are DDI alerts, drug–allergy alerts, recommended doses, and drug information when prescribing any medication to a patient (Bell et al., 2019; Kawamoto et al., 2005).

From Table 1, only the Surescripts (i.e., the US e-Prescription network) and Spain's e-Prescription systems have DDI alerts integrated in their systems (Ministry of Health, Social Services and Equality, 2014; Surescripts, 2018a). For other countries, to the best of our knowledge, there is no mention on the systems' websites about the description of their system, or the system architecture does not have the required CDS features.

However, other survey studies suggested that most systems are likely to incorporate the CDS. For example, in the United Kingdom, CDS systems are not a part of hospitals' systems or part of the e-Prescription system, but there is interoperability between the CDS systems and other systems to help with prescribing medications to patients safely [Bell et al., 2019; Health and Social Care Information Centre (Great Britain), 2019; Ojeleye et al., 2013]. Moreover, a survey on the most common methods used to identify any case of Potential of Drug–Drug Interactions (PDDI) found that more than half of the participants tend to search for the drug name and use facts and comparisons to identify PDDI. They used various keyword strategies to search for multiple databases and web resources (Grizzle et al., 2019).

The patient's medication history is an essential part of improving the safe prescription of medication to a patient. This feature is likely to help avoid any DDI and enhance the treatment process to lead to personalized care (Blouin and Adams, 2017; Bush and Daniels, 2017; Nester and Hale, 2002).

In Table 1, we see that not all the systems have this feature available to the prescriber. However, most of the systems incorporate this feature in EHR systems. For example, the UK system has this information in the patient record rather than in the e-Prescription service. The incorporation of medication history is different in some countries because of their definition of the e-Prescription system. In the United Kingdom, e-Prescription is defined as a service for transferring electronic prescriptions from a prescriber to a pharmacy. While in Japan, the medication history information is included in a patient's e-Prescription service application (Ministry of Health, Labor and Welfare, 2016).

Moreover, to save doctors' time, a new approach was proposed for displaying patients' medication history in a timeline model. In their timeline, the medications will be displayed relevant to the time a patient took them. Their design aims to provide a better understanding of a patient's complex medication history, which is likely to help a prescriber reduce the work rate load of looking up the medication history and when those medications are taken.

Issuing an e-Prescription for controlled medication is a significant limitation in all the systems mentioned above, except Surescripts (2018b). In the United States, the e-Prescribing of controlled medication was permitted in 2010, and the certification process was approved in 2013 (Drug Enforcement Administration, 2010, 2013). In other systems, to the best of our knowledge, there is no available information about how to use e-Prescriptions to dispense controlled medication or the e-Prescription service does not offer the prescription of controlled medication.

### Patient identity verification and e-prescription encryption

The need for a unique ID for all the involved parties in e-Prescription systems is crucial to make the systems fully automated. We can see in Table 2 that most of the systems have assigned unique IDs for the involved parties in the system, that is, patient ID, prescriber ID, and pharmacy ID. Assigning unique IDs for the abovementioned parties is likely to help manage to transfer e-Prescriptions efficiently and help avoid transferring or storing errors. Moreover, assigning unique IDs to each prescription and medication is likely to help manage each patient's prescription and all the prescribed medications in that prescription. As a standard practice, prescription IDs and medication IDs were used to keep a medication record for each patient at the pharmacy. Furthermore, prescriptions and medication records help manage the vast number of prescriptions a pharmacy had to manage.

**Table 2.** Comparison of Security and Privacy Features Across the Systems

System	Surescripts	PrescritbeIT	United Kingdom	Sweden	Denmark	Spain	Australia	Japan
Pharmacy ID	✓	—	✓	×	×	—	✓	×
Prescriber ID	✓	—	✓	✓	✓	—	✓	×
Medication ID	✓	—	✓	✓	✓	✓	✓	✓
Prescription ID	×	✓	✓	×	×	✓	✓	✓
Patient ID	Master index	✓	✓	✓	✓	✓	✓	✓
Patient ID verification	—	—	—	—	—	Health card	—	×
Participate consent	×	×	Choosing pharmacy	×	×	—	✓	×
Using HL7^a^	✓	—	✓	×^b^	×^c^	—	✓	×

^a^ HL7 is communication protocol to transfer the medical information from ehealth service system to another. HL7 is used to encode the information to be readable to all the ehealth service systems (Bender and Sartipi, 2013; HL7, 2019; Saripalle et al., 2019).

^b^ Sweden eHealth systems uses a service-oriented communication endpoint for the technical protocol, They use ENV 13607 standard (Doupi et al., 2010; Mäkinen et al., 2011; Sellberg and Eltes, 2017).

^c^ Denmark e-Prescription uses the MedCom communication standard nationwide. MedCom was established in 1994 to develop the communication standards for transferring the medical records and information between health centers nationwide (Krag et al., 2012; Öhlund et al., 2012).

HL7, Health Level Seven International.

Despite all the unique IDs used in the e-Prescription systems mentioned in Table 2, to the best of our knowledge, there is no evidence from their websites that they are using them in their e-Prescription system to verify patients' identities in the medication dispensing process. In Spain's e-Prescription system, the patient is required to show their health card to pick up their medication. However, other verification methods might be in place (e.g., asking for the patient's name, birthday, address).

In terms of the communication protocol, most of the systems are using the HL7 protocol to encode and decode e-Prescription information between the involved parties (Chen et al., 2016; Chouvarda and Maglaveras, 2015; Eichwald, 2014; Goundrey-Smith, 2013; Pereira et al., 2018; Saripalle et al., 2019). For encryption, most systems use standard encryption methods such as public key infrastructure such as in Australia and Canada (Canadian Pharmacists Association, 2009; Henderson et al., 2015), or other standard authentication algorithms.

## Discussion

### Overarching context and related work

A comparative study between five countries (United States, United Kingdom, Sweden, Denmark, and Finland) has compared the e-Prescription systems in those countries (Samadbeik et al., 2017). The latter study aimed to evaluate and compare the available e-Prescription systems in the selected countries. The review period of the study was 2013–2015. The authors had three phases of selecting the participating countries in the study. First, they selected all the countries with a fully implemented e-Prescription system such as the EU and United States. In the second phase, they eliminated the countries that did not fit their specified criteria regarding their proposed system's preferred features.

The authors chose three features that must be in the definition of e-Prescription. The features were, namely, electronically creating e-Prescriptions, electronically sending the e-Prescriptions to the pharmacy from the prescriber, and two-way communication between the pharmacy and the prescriber. This study limited the selection to eight countries (Denmark, Finland, Germany, New Zealand, the Netherlands, Sweden, United Kingdom, and the United States), which have the potential to have the specified features mentioned above. The final stage of selecting the participating countries was to review the national prescription system in each country. The authors aimed to select only the systems capable of electronically sending prescriptions to the pharmacies and providing a two-way communication channel between the prescriber and the pharmacist.

As a result, only five of the initially selected countries were eligible for the review study. Later, they created a data collection form from the main components of the prescription system model. They collected the data using the search engines and related websites of the selected countries' e-Prescription service. Moreover, they sent emails to the organizations that provide the e-Prescription service to clarify any ambiguity regarding the information collected about the service (Samadbeik et al., 2017). They categorized the results regarding the main components of the e-Prescription service model.

First, they found in all the selected countries that the prescriber's electronic signature was required and legal. Also, the consent of the patient is necessary to access the required information from the involved parties. However, none of the countries accept or process e-Prescriptions from other countries.

Second, in the comparison results about the e-Prescription systems' architecture, they found that all the European countries use a centralized system and have a national database of e-Prescriptions. However, in the United States, the system is decentralized and not controlled or managed by a national organization, which results in the absence of an e-Prescription national database. The rest of the countries use governmental resources to provide e-Prescription services.

Third, in terms of setting identification information for prescriptions and patients, the United States, Sweden, and Denmark do not have a Prescription Unique ID (PUID) at the time of creating the prescription. The PUID is used to link the prescriptions to the patients and help to keep records of past ones. In addition, only the US system does not have patient identification information, used to identify patients in the database. Finally, only the US e-Prescription system provides pharmacies' ability to request the patient's historical information from the prescriber. To implement a fully functioning e-Prescription system, the authors concluded that a country needs to have the base infrastructure for the e-health and national e-Prescription database (Samadbeik et al., 2017).

Another study focused on examining the economic, health, and social benefits gained from e-Prescription systems across Europe. Their findings confirmed that e-Prescriptions would benefit the involved parties in the e-Prescription systems economically. Such benefits are cost savings from the level of transparency provided by the system, reducing the fraud related to the systems and minimizing the cost of printing prescriptions. In terms of health benefits, the system reduced medication errors, provided a better level of medicine accessibility, and improved the monitoring of patient medication intake. Furthermore, the system's leading social benefit is the increased confidence of the patient toward the prescribing system (Deetjen, 2016). However, those benefits will depend on the country's e-Prescription system architecture and its implementation process.

A review was conducted on the literature and government reports relevant to implementing e-Prescribing systems at a national level in several European countries (Kierkegaard, 2013). They aimed to examine the issues that will limit providing eHealth services across EU countries' borders. The study found that the EU countries have different health care policies, different levels of medical data privacy laws, communication networks and methods between the involved parties in e-Prescription systems, and various implementations of the prescriber's digital signature for e-Prescriptions. From the findings, the authors stated that the interoperability of different eHealth systems across the EU countries is part of the solution. More importantly, the authors opined that medical data's privacy and security should be enforced equally among the EU countries (Kierkegaard, 2013).

A recent study was conducted in Finland to explore the e-Prescription anomalies (i.e., errors, ambiguities, and other shortcomings) frequency occurrence, what methods to clarify the e-Prescription, and how those anomalies affect the patient safety in the community pharmacies. Of the surveyed nearly 41,000 e-Prescriptions during the study period (i.e., 3 days), only 7% of the dispensed e-Prescription had anomalies. A total of 54 community pharmacies, who participated in the study, reported those anomalies. Almost 63% of the e-Prescriptions contained errors in the dosage intake instruction (i.e., the most common anomalies), and 28% of the e-Prescriptions were missing the reason for using the prescribed medication. In most of the 69% anomalies cases, the pharmacist clarified them by writing the dosage instructions, and nearly 23% of them, the patient corrected the dosage instructions.

Accordingly, the pharmacy's workload will increase from interpreting the pharmacist's e-Prescriptions' anomalies, which will affect the overall quality of service. In the above anomalies cases, the pharmacy's workload increased by 39%, which led to an increase in the wait time for the patient (Timonen et al., 2018).

Table 3 shows the scope of the current study results compared with the previous studies. In this study, we expanded the scope of studied countries to get a global overview of a number of the leading countries in e-Prescription. Moreover, we believe that expanding the scope and exploring the implemented systems is more likely to help adopt new approaches to implement more efficient digital health systems, specifically e-Prescription systems in the future. Furthermore, our study aims to compare the security and privacy protocols in place for the selected countries and the system architecture. Moreover, we evaluate the capabilities of the surveyed countries to adopt new technologies, specifically Blockchain and AI. Finally, the study proposes solutions from a technical view to overcoming the resultant challenges and limitations.

**Table 3.** Comparison of Previous Studies and the Current Study

		System architecture	Medication history	CDS	Patient privacy	System security	AI and Blockchain capability
Previous studies (Deetjen, 2016; Kierkegaard, 2013; Samadbeik et al., 2017; Timonen et al., 2018)	Canada	XC	XC	XC	XC	XC	XC
	United States	D	✓	✓	XA	XA	XA
	United Kingdom	C	✓	✓	XA	XA	XA
	Spain	XC	XC	XC	XC	XC	XC
	Denmark	C	✓	✓	XA	XA	XA
	Sweden	C	✓	✓	XA	XA	XA
	Australia	XC	XC	XC	XC	XC	XC
	Japan	XC	XC	XC	XC	XC	XC
Current study	Canada	C	✓	✓	✓	✓	AI
	United States	D	✓	✓	✓	✓	X
	United Kingdom	C	✓	✓	✓	✓	AI
	Spain	C	✓	✓	✓	✓	AI
	Denmark	C	✓	✓	✓	✓	X
	Sweden	C	✓	✓	✓	✓	X
	Australia	D	✓	✓	✓	✓	AI
	Japan	D	✓	✓	✓	✓	X

AI and Blockchain capability: AI, the infrastructure for AI exist; X, not ready.

AI, artificial intelligence; C, centralized system; CDS, clinical decision support; D, decentralized; XA, information about the aspect not included in the study; XC, the country is not included in the study; ✓, information about this aspect was included in the study.

### Limitations and challenges

After exploring the current e-Prescription systems, it is clear that they are different in applying this service. The difference is due to several reasons; some related to the countries' regulations and rules or the existing infrastructure (Samadbeik et al., 2017). However, several limitations might hinder the progress of improving the quality of the service provided to the patient.

Centralized or decentralized systems are progressing toward applying the Internet of Things (IoT) solutions in health care services to enhance the quality of service and efficiency regarding the provided service. Moreover, an essential factor when handling a patient's medical information is the privacy and security of their medical data. E-Prescription and medication history are part of the patient's medical data. This part of medical data requires a critical level of privacy, and it should be stored securely due to the severe risks associated with it.

One type of risk is tampering with a patient's medication intake instructions, which could cause the patient's death. Therefore, many researchers emphasize the need for security and privacy policies and protocols to use IoT solutions in health care (Al-Nayadi and Abawajy, 2007; Azad et al., 2019; Ball et al., 2003; Park and Moon, 2016). One crucial challenge of e-Prescription systems is whether the system's overall architecture should be centralized or decentralized. As shown in Table 1, many e-Prescription services are centralized and connect to the patients' EHR system. Moreover, some countries have adopted the decentralized approach because of the existing infrastructure. For example, in the United States, EHR systems are available at most hospitals and health care centers.

Although the US system is a decentralized system for e-Prescription, it is still a network that facilitates communication between the involved parties. Surescript is heavily dependent on the local centralized system in the health care center or the pharmacies to store their patient data. Therefore, the network will more likely be vulnerable to the security threats caused by the centralized system connected to it. Moreover, from Table 1, we can see that most of the decentralized systems are dependent on centralized local systems and that it is to facilitate the process of collecting medical data. In Australia, their e-Prescription service is connected to the main EHR system, which is centralized. However, Japan's e-Prescription service uses the patient's mobile application to store the patient's medication history. Therefore, Japan is the most decentralized e-Prescription service compared with the United States and Australia.

Because of the issues related to centralized systems, several novice approaches proposed decentralized systems for health records, medication histories, and e-Prescription to preserve patients' privacy and prevent any pointed attacks on medical information (Li et al., 2019).

However, many countries' regulations require a central physical location to control access to medical data. Therefore, an adaptable approach is likely to help solve most of the architecture issues, such as a system designed to store, transfer, and share needed data (e.g., the prescription history or medication history of a patient) from the patient. Such a system can use any authentication protocol through a token handed to the patient, a key stored in a barcode, or a mobile application accessed by only the patient. From Table 2, we can see that the United States and Australia have most of the needed identifiers to facilitate the management of the required data about medications and e-Prescriptions. These systems are more likely to adopt a new approach toward using Blockchain, which will be more likely to protect against the security threats related to centralized systems.

#### Medication history

Another challenging issue is the availability of medication histories to other parties participating in the system, such as pharmacists. A quantitative study about the differences between medication histories obtained by physicians and pharmacists was conducted by reviewing 200 medical records. The authors found that pharmacists are better at identifying medication information from patients' medication histories than physicians (Hatch et al., 2011). In addition, several studies found that information of medication histories collected by pharmacists' interviews are complete when compared with the information collected by other caregivers (Carter et al., 2006; LaPointe and Jollis, 2003; Tam et al., 2005; Vira et al., 2006).

As a result, making the medication histories available to all parties involved in the system might enhance patient safety when prescribing or dispensing a new medication. Moreover, other study results showed that caregivers collect medication history information from patients at the initial interview during the admission process (Nester and Hale, 2002). This process makes the information unreliable due to human errors, as it is dependent on the patient's memory, and it can lead to inaccurate information (Hatch et al., 2011).

Thus, having electronic medication histories available and accessible to transfer when needed can improve the efficiency and quality of the provided services. Canada, the United States, Spain, Australia, and Japan are progressing well by making the medication history available to all participating parties. However, only Spain looks like it is ready for adopting future technologies. Despite the United States having the ability to share the medication history, it is still a slow process that needs to be accelerated to make the medication history available in case of emergency. Moreover, the United States, Canada, Australia, and Japan make the medication history available as a service depending on the data stored in the health care centers and pharmacies. This is more likely to slow the progress to adopting AI technologies. A dedicated server to collect and process the data is more likely to help toward that.

#### Clinical decision support

CDS systems are developed to help prescribers prescribe medications safely and alert them of the various drug interactions that might occur while prescribing a medication to a patient (Bell et al., 2019). Many studies showed an improvement in avoiding medication errors when using e-Prescription with CDS alerts (Ammenwerth et al., 2008; Cresswell et al., 2014; Eslami et al., 2007; Kaushal et al., 2003; Prgomet et al., 2016). However, other studies showed that prescribers tend to ignore and override less important alerts when overwhelmed by a number of alerts and how the system is displaying them (Embi and Leonard, 2012; Van Der Sijs et al., 2006).

When the number of less important alerts increases, this might increase the risk of medication errors. Additionally, the authors found in their systemic review that between 49% and 96% of drug interaction alerts were overridden or ignored (Van Der Sijs et al., 2006). Therefore, incorporating CDS alerts to an e-Prescription system is a necessity, and new visualization methods could reduce the ignoring and overriding of cases.

In addition, a new algorithm based on the patient's medication information might reduce the number of less important alerts. The United States and Spain are the only systems that provide this service as part of the e-Prescription system. However, the CDS systems are progressing toward using AI technologies to enhance the patient's quality of care. Therefore, a large amount of data collection is needed for this progress, which from Table 1 shows Spain is leading the score. US system needs to progress toward data collection and processing to meet the new demands of a better quality of care.

### Proposed solutions

#### Blockchain

Blockchain is a technology deployed best for decentralized systems. It is a technology to store the data in a secure and distributed method. This technology intended to remove the need for a centralized authority to control and verify the data (Li et al., 2019). Therefore, we can see from Tables 1 and 3 that the United States, Australia, and Japan are candidates to implement the Blockchain method because of their decentralized systems. However, those systems still lack the connection between the other parties to facilitate such an approach.

In the United States, e-Prescription systems are handled by a middleman (i.e., Surescripts), making the system semicentered when it comes to managing data sharing between the subscribers (Li et al., 2019). As mentioned previously, the Surescripts enables subscribers to request patient records from other health centers. Other centers will then handle the request, and they have the option to share or hold that information (Surescripts, 2019). This process is more likely to limit the progress toward integrating Blockchain technology. The Blockchain aims to store the data securely and make the data available to all the involved parties.

Australia and Japan's e-Prescription systems are not a fully decentralized system, and their approach is to provide peer-to-peer communication between the prescriber and the pharmacy. This approach allows the pharmacies to send an update to the prescriber system about their patients' e-Prescriptions. Therefore, the infrastructure of those systems lacks the capability at this time of adopting Blockchain technology.

Regarding the centralized systems, adopting the technology is more challenging since their approach is to have a central point to control the information. This approach is more costly to provide the needed security and privacy to protect patient data. Installing and managing patient data security might cost hundreds of millions of dollars (Becker's Healthcare, 2016; Li et al., 2019). Thus, an approach containing more of the benefits of the decentralized architecture integrated with Blockchain will save costs to manage the patients' security and privacy data. Moreover, this approach more likely helps save valuable time wasted to look up the updated medication history of a patient (Norén et al., 2008; Schmiedl et al., 2014).

#### Artificial intelligence

AI in health care is introduced to support the medical decision. AI is more likely to be adopted as the next logical step in health care technologies. It is more likely to provide better patient care knowledge and keep updated information about patient status. ML and Deep Learning (DL) are the leading technologies in AI. Both technologies are developed to learn patterns about a type of information to suggest accurate predictions. For the system to predict efficiently and accurately, these technologies require learning patterns from large amounts of data. Thus, the type and size of collected data about a patient are important factors. The infrastructure to collect the data is key to assessing the capability of the surveyed systems (Flynn, 2019).

Therefore, we can see from Table 1, the leading country of collecting data is Spain. The type of collected data in Spain's system is an essential factor and more likely to help adopt the ML and DL faster than other countries. However, the communication between parties in Spain might limit this process, as shown in Table 2. On the other hand, the centralized systems are more likely to adopt these technologies faster than the decentralized systems (e.g., United States) due to the required data collection process.

To summarize, it may be worthwhile to consider a different e-Prescription model to overcome the discussed challenges in the current systems. This model should include the ability to share prescription and medication history information between all participating parties in the system. This approach could benefit from the available centralized systems in the countries by incorporating a standalone service that transfers and stores medication history data and e-Prescriptions securely. This service should also preserve the patients' privacy by applying an authentication mechanism to the authorized parties so they can access the data such as Blockchain. Moreover, medication histories should be kept available to patients to enhance patient safety regarding medication errors. Also, this process will grant the patient the ability to share accurate medication histories.

Lastly, CDS systems should be incorporated in the e-Prescription service and also redesigned to avoid ignoring and overriding alert issues when the less important alerts overwhelm the caregiver. In addition, redesigning the system to incorporate future technologies such as AI technologies will more likely enhance the care quality of the patient. Furthermore, in the current COVID-19 climate, e-Prescription systems have become highly relevant in preventing unnecessary contact and ensuring patient and caregivers' safety.

## Conclusions

In this study, we compared the selected e-Prescription systems. The comparison process is based on the systems' security and privacy protocols and the systems' architecture. Furthermore, we evaluated the systems' capabilities to progress toward using future technologies such as Blockchain and AI. Finally, we believe this survey provides broad and timely insights on e-Prescription systems around the world. We suggest conducting future studies about the capabilities of the e-Prescription systems to cooperate and communicate on a global scale. This research might contribute toward designing a universal e-Prescription system design that is available to patients when traveling outside of their home country.

## Abbreviations Used

ADR: Adverse Drug Reactions
AI: Artificial intelligence
CDS: clinical decision support
DDI: Drug-Drug Interactions
DIS: Drug Information System
DL: deep learning
EHR: Electronic Health Record
EMR: Electronic Medical Record
EPS: Electronic Prescription Service
eRx: electronic medical prescription
ETP: Electronic Transfer Prescription
EU: European Union
FHIR: Fast Healthcare Interoperability Resources
HL7: Health Level Seven International
IoT: Internet of Things
ML: machine learning
NHS: National Health Service
PA: prior authorization
PBM: pharmacy benefit manager
PDDI: Potential of Drug–Drug Interactions
PES: Prescription Exchange Service
PHR: Personal Health Record
PUID: Prescription Unique ID
